# Nanoparticles (NPs)-mediated lncMALAT1 silencing to reverse cisplatin resistance for effective hepatocellular carcinoma therapy

**DOI:** 10.3389/fphar.2024.1437071

**Published:** 2024-07-30

**Authors:** Ting Wang, Qianyao Li, Rui Xu, Zixuan Zhao, Qi Sun, Xiaoding Xu, Rong Li

**Affiliations:** ^1^ The Second Affiliated Hospital, Department of Pharmacy, Hengyang Medical School, University of South China, Hengyang, China; ^2^ Guangzhou Key Laboratory of Medical Nanomaterials, Sun Yat-Sen Memorial Hospital, Sun Yat-Sen University, Guangzhou, China; ^3^ Nanhai Translational Innovation Center of Precision Immunology, Sun Yat-Sen Memorial Hospital, Foshan, China; ^4^ Department of Neurosurgery, Yiyang Central Hospital, Yiyang, China

**Keywords:** chemotherapy, resistance, lncRNA, nanoplatform, co-delivery

## Abstract

Platinum-based chemotherapy has been widely used for clinical cancer treatment, but drug resistance is the main barrier to induce the poor prognosis of cancer patients. Long non-coding RNAs (lncRNAs) have been recognized as a type of new cancer therapeutic targets due to their important role in regulating cancer progression such as drug resistance. However, it is still challenged to effectively intervene the expression of lncRNAs as they are usually located at various subcellular organelles (*e.g.*, nucleus, mitochondrion, and endoplasmic reticulum). We herein developed an endosomal pH-responsive nanoparticle (NP) platform for small interfering RNA (siRNA) and cisplatin prodrug co-delivery and effective cisplatin-resistant hepatocellular carcinoma (HCC) therapy. This co-delivery nanoplatform is comprised of a hydrophilic polyethylene glycol (PEG) shell and a hydrophobic poly (2-(diisopropylamino)ethyl methacrylate) (PDPA) core, in which cisplatin prodrug and electrostatic complexes of nucleus-targeting amphiphilic peptide (NTPA) and siRNA are encapsulated. After intravenous injection and then uptake by tumor cells, the endosomal pH could trigger the dissociation of nanoplatform and enhance the endosomal escape of loaded cisplatin prodrug and NTPA/siRNA complexes via the “proton sponge” effect. Subsequently, the NTPA/siRNA complexes could specifically transport siRNA into the nucleus and efficiently reverse cisplatin resistance via silencing the expression of lncRNA metastasis-associated lung adenocarcinoma transcript 1 (lncMALAT1) mainly localized in the nucleus, ultimately inhibiting the growth of cisplatin-resistant HCC tumor.

## Introduction

Cancer is the main leading cause of death globally, accounting for around 9.7 million deaths in 2022 (Bray et al., 2024). In past few decades, a variety of therapeutic modalities such as chemotherapy, molecular targeted therapy, radiotherapy, and immunotherapy have been developed for cancer treatment. Among them, platinum-based chemotherapy is the main therapeutic modality for the treatment of various types of cancers (Wang and Lippard, 2005; Rottenberg et al., 2021; Zhang et al., 2022b). However, drug resistance is the main barrier to induce the poor prognosis of cancer patients (Ward et al., 2021; Emran et al., 2022; Shi et al., 2023). More importantly, accumulating evidences have revealed that cancer patients usually develop resistance rapidly to other chemotherapeutic drugs (*i.e.*, multidrug resistance) once they are resistant to a certain chemotherapeutic drug (Li et al., 2020; Shi et al., 2023). Currently, chemoresistance has been recognized as one of the hallmarks of cancer and how to effectively overcome chemoresistance has long been a key issue to be solved (Cree and Charlton, 2017; Hanahan, 2022; Xia et al., 2024). Therefore, understanding the inherent mechanisms regulating chemoresistance is of utmost importance for the development of effective strategy to reverse chemoresistance and enhance therapeutic outcomes.

Long non-coding RNAs (lncRNAs) are a type of RNA transcripts with a structural similarity as messenger RNA (mRNA) and account for more than 50% of human genome transcripts (Ulitsky and Bartel, 2013; Slack and Chinnaiyan, 2019; Statello et al., 2021). Initially, lncRNAs had been recognized as by-products transcribed by RNA polymerase II since they do not encode proteins. However, as the continuous deepening of molecular biology research, overwhelming evidences have revealed that lncRNAs can regulate the expression of target genes at both transcriptional and post-transcriptional levels, thus widely involving in the development and progression of various diseases including cancer (Anastasiadou et al., 2018; Slack and Chinnaiyan, 2019; Saw et al., 2021). In the past decade, with the emergency and rapid development of high-throughput sequencing technology, numerous functional lncRNAs have been uncovered, which could be used as predictors for the prognosis of cancer patients and/or therapeutic targets for cancer therapy (Wahlestedt, 2013; Liu et al., 2021; Yang et al., 2023). For example, lncRNA metastasis-associated lung adenocarcinoma transcript 1 (lncMALAT1) and plasmacytoma variant translocation 1 (lncPVT1) are the two representative lncRNAs, and their high expression can induce resistance to various the therapeutic modalities (*e.g.*, chemotherapy) and promote the recurrence and metastasis of a variety of cancer types, such as breast, liver, and lung cancer (Schmitt and Chang, 2016; Hou et al., 2023; Zhang et al., 2023). For example, a recent study has revealed that lncMALAT1 could enhance the expression of Aurora-A via the interaction with miR-140-5p to induce resistance to sorafenib (Fan et al., 2020). Based on the important role of lncRNAs in cancer development and progression, targeted regulation of their expression has been considered as a promising strategy for effective cancer therapy. Unfortunately, unlike cancer-associated proteins whose activity can be blocked by using various tools such as inhibitors and antibodies, it is still challenged to effectively regulate the expression of lncRNAs (Wahlestedt, 2013). At present, nucleic acids including small interfering RNA (siRNA) and plasmid DNA are the most effective tool due to their specific characteristic of regulating the expression of any target genes (Kulkarni et al., 2021; Zhu et al., 2022). However, as negatively charged biomacromolecules, nucleic acids can be easily degraded by nuclease and cannot cross cell membrane (Mendes et al., 2022; Paunovska et al., 2022). More importantly, lncRNAs are usually localized at various subcellular organelles (*e.g.*, nucleus, mitochondrion, and endoplasmic reticulum) (Yao et al., 2019; Bridges et al., 2021; Wei et al., 2023), which could thus bring additional difficulty in regulating their expression by nucleic acids.

**FIGURE 1:**
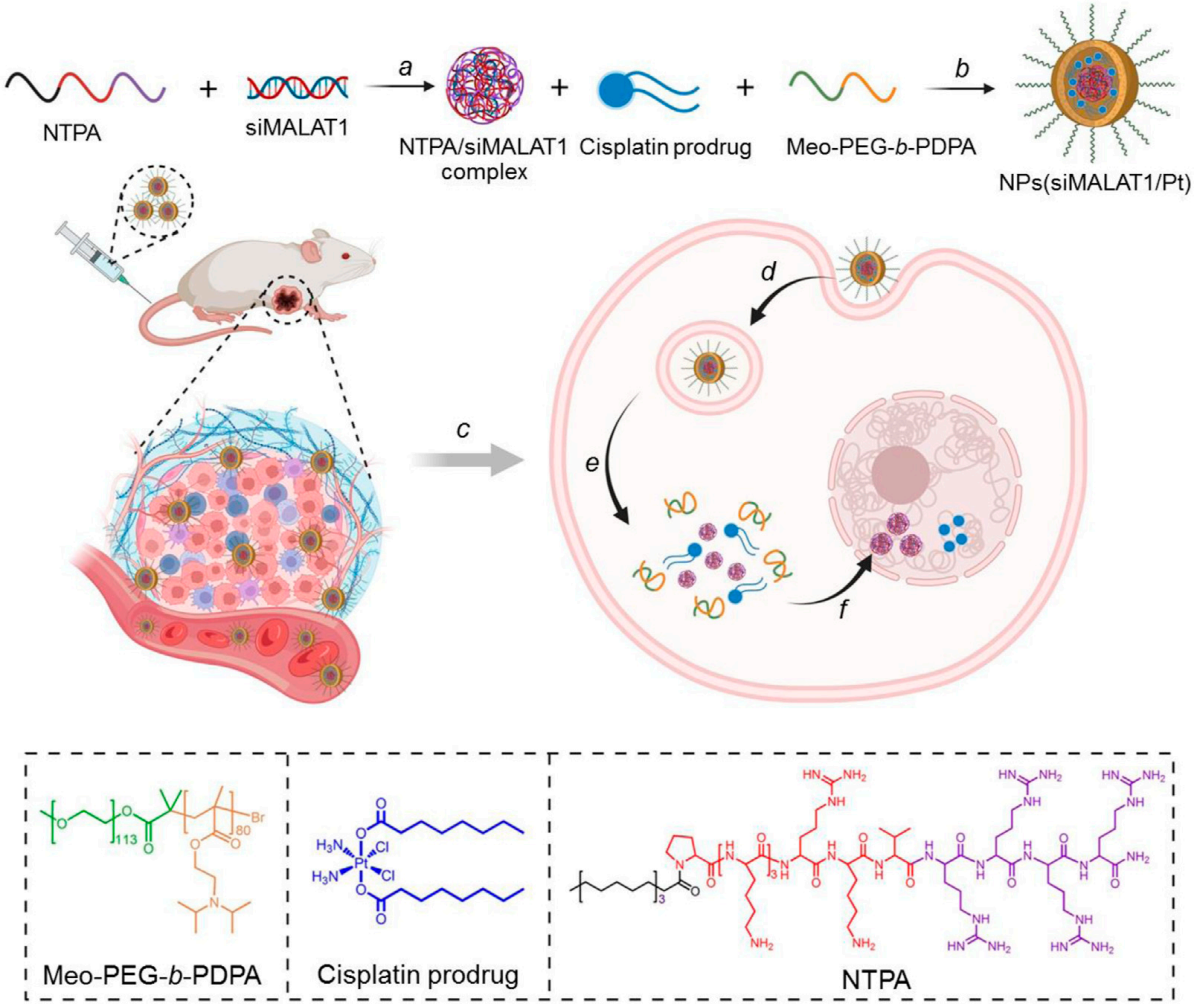
Schematic illustration of the endosomal pH-responsive nanoplatform (*i.e.*, NPs(siMALAT1/Pt)) to co-deliver siMALAT1 and cisplatin prodrug for effective cisplatin-resistant HCC therapy. This nanoplatform is comprised of a hydrophilic PEG shell and a hydrophobic PDPA core that could encapsulate cisplatin prodrug and NTPA/siMALAT1 complexes **(A, B)**. After intravenous injection **(C)** and internalization by tumor cells **(D)**, the endosomal pH could trigger the disassociation of nanoplatform into free cisplatin prodrug and NTPA/siMALAT1 complexes **(E)**. Subsequently, the NTPA/siMALAT1 complexes could specifically transport siMALAT1 into the nuclei and significantly silence lncMALAT1 expression **(F)**, ultimately leading to the reversal of cisplatin resistance and significant inhibition of cisplatin-resistance HCC tumor.

To address these issues, we herein developed an endosomal pH-responsive nanoparticle (NP) platform to co-deliver siRNA and glutathione (GSH)-responsive cisplatin prodrug for lncMALAT1 silencing and effective cisplatin-resistant hepatocellular carcinoma (HCC) therapy (Figure 1). This co-delivery nanoplatform is comprised of a hydrophilic polyethylene glycol (PEG) shell and a hydrophobic poly (2-(diisopropylamino) ethyl methacrylate) (PDPA) core, in which cisplatin prodrug and electrostatic complexes of nucleus-targeting amphiphilic peptide (NTPA) and siRNA are encapsulated. After intravenous administration and then internalization by tumor cells, the endosomal pH could trigger the dissociation of nanoplatform and enhance the endosomal escape of loaded cisplatin prodrug and NTPA/siRNA complexes via the “proton sponge” effect (Yu et al., 2011). Subsequently, the NTPA/siRNA complexes could specifically transport siRNA into the nuclei (Goldfarb et al., 2004; Pouton et al., 2007; Huang et al., 2022) and efficiently reverse cisplatin resistance via silencing of lncMALAT1 expression. Combining the anticancer effect of cisplatin obtained via the reduction of cisplatin prodrug by the highly concentrated GSH in the cytoplasm of HCC cells (Shen et al., 2018; Ling et al., 2019; Zhang et al., 2022a), the co-delivery nanoplatform could significantly inhibit the growth of cisplatin-resistant HCC tumor.

## Materials and methods

### Materials

Nucleus-targeting peptide-amphiphile (NTPA, sequence: C_17_H_35_CONH-PKKKRKVRRRR-OH) was obtained from GL Biochem Ltd. Amphiphilic copolymer methoxyl-polyethylene glycol-b- poly (2-(diisopropylamino) ethyl methacrylate) (Meo-PEG-*b*-PDPA) and GSH-responsive cisplatin prodrug were synthesized according to our previous reports (Li et al., 2020; Yang et al., 2023). The siRNA targeting lncMALAT1 was acquired from IGE Bio. The siRNA sequences are as follows: siMALAT1, 5′-GAG CAA AGG AAG UGG CUU ATT-3’ (sense) and 5′-UAA GCC ACU UCC UUU GCU CTT-3’ (antisense). Cy5-siMALAT1 was purchased from IGE Bio and Cy5 was labeled at the 5′-end of both the sense and antisense strands. The primers for reverse transcription quantitative polymerase chain reaction (qRT-PCR) are as follows: lncMALAT1, 5′-GCT TGA GAA GAT GAG GGT GTT T-3′ (forward sequence), 5′- TCC AAA AGC CTT CTG CCT TA-3′ (reverse sequence); GAPDH, 5′-AGG TCG GTG TGA ACG GAT TTG-3′ (forward sequence), 5′-TGT AGA CCA TGT AGT TGA GGT CA-3′ (reverse sequence). Ki67 rabbit mAb (#ab92742) and anti-rabbit IgG horseradish peroxidase (HRP)-linked secondary mAb (#7074) were respectively purchased from Abcam and Cell Signaling Technology (CST). Click-iT™ TUNEL Colorimetric IHC Detection Kit (#C10625) was obtained from ThermoFisher. Dulbecco’s Modified Eagle Medium (DMEM), penicillin-streptomycin, trypsin, and fetal bovine serum (FBS) were purchased from Invitrogen Corp. and used received. All other reagents and solvents are of analytical grade and used without further purification.

### Preparation and characterizations of NPs

The co-delivery NPs were prepared using a modified nanoprecipitation method (Valencia et al., 2010; Li et al., 2020). Briefly, *N,N′*-dimethylformamide (DMF) solution of Meo-PEG-*b*-PDPA (20 mg/mL, 200 μL), NTPA (5 mg/mL, 50 μL), and cisplatin prodrug (20 mg/mL, 20 μL) were mixed with 10 μL of siMALAT1 aqueous solution (0.1 nmol/μL). Under vigorously stirring (1,000 rpm), the mixture was added dropwise to 5 mL of deionized water. The formed NP suspension was subjected to an ultrafiltration device (EMD Millipore, MWCO 100 K) and centrifuged to remove the organic solvent and free compounds. After washing with phosphate buffered saline (PBS) solution, the obtained co-delivery NPs (denoted NPs(siMALAT1/Pt)) were suspended in 1 mL of PBS solution. The NPs only loading siMALAT1 (denoted NPs(siMALAT1)) or loading scramble siRNA and cisplatin prodrug (NPs(siCTL/Pt)) were prepared according to the method described above. In addition, Control NPs were also prepared by changing NTPA with cationic lipid-like compound G0-C14 we previously developed (Li et al., 2020; Yang et al., 2023). Size and zeta potential were determined by dynamic light scattering (DLS, Malvern, USA). The morphology of NPs was visualized under a transmission electron microscope (TEM) (Tecnai G^2^ Spirit BioTWIN). To determine the EE% of siRNA and cisplatin prodrug, Cy5-siMALAT1 was encapsulated into the NPs (denoted NPs(Cy5-siMALAT1/Pt)) and the fluorescence intensity of Cy5-siMALAT1 was measured using a Synergy HT multi-mode microplate reader (BioTek, USA). At the same time, the NPs were subjected for inductively coupled plasma mass spectrometry (ICP-MS) analysis of platinum content. The EE% of siRNA (∼71.6%) and cisplatin prodrug (∼39.8%) is calculated by comparing to standard curve.

### Cell culture

Cisplatin-resistant HCC cells (Bel7402-DR) were incubated in DMEM containing 10% FBS and 2 mg/L cisplatin at 37 ^o^C in a humidified atmosphere containing 5% CO_2_. The parental HCC cells (Bel7402-DS) were incubated in DMEM with 10% FBS at the same conditions.

### Endosomal escape and entry of nucleus

Bel7402-DR cells (50,000 cells) were seeded in round discs and incubated in 2 mL of DMEM containing 10% FBS for 24 h. Subsequently, the NPs(Cy5-siMALAT1/Pt) were added at a siRNA dose of 30 nM and the cells were allowed to incubate for different times. After removing the medium and then respectively staining the endosomes and nuclei with Lysotracker green and Hoechst 33342, the cells were viewed under a ZEISS 800 confocal laser scanning microscope (CLSM). After the observations, the cells were trypsinized and the nuclei were collected using Ambion PARIS (Life Technologies) according to the manufacturer’s protocol. After repeated freezing in the liquid nitrogen and then thawing at room temperature, the top solution was collected and the fluorescence intensity of Cy5-siMALAT1 in the nuclei was examined using a microplate reader.

### *In Vitro* LncMALAT1 silencing

Bel7402-DR cells (50,000 cells) were seeded in 6-well plate and incubated in 2 mL of DMEM containing 10% FBS for 24 h. Subsequently, the NPs(siMALAT1/Pt) were added at different siRNA doses and then incubated with the cells for 24 h. After washing with PBS (pH 7.4) solution, the cells were further incubated in fresh medium for another 48 h. Thereafter, total RNA in the cells was extracted from the cells using Trizol and lncMALAT1 expression was examined using qRT-PCR. To directly observe lncMALAT1 expression in Bel7402-DR cells, the cells (50,000 cells) were seeded in a round disc and then treated with the NPs(siMALAT1/Pt) according to the aforementioned protocol. Thereafter, the cells were fixed with 4% paraformaldehyde (PFA) and lncMALAT1 expression was observed under a ZEISS 800 CLSM after staining lncMALAT1 with fluorescence probe according to the standard protocol of fluorescence *in situ* hybridization (FISH).

### Apoptosis detection

Bel7402-DR cells were seeded in 6-well plates (50,000 cells per well) and treated with the NPs(siMALAT1/Pt) at a siRNA dose of 30 nM according to the method described above. The cells were then trypsinized and collected for PI PE and FITC Annexin V staining using PE Annexin V Apoptosis Detection Kit. The apoptosis analysis was performed using a DXP11 Flow Cytometry Analyzer.

### *In Vitro* cell proliferation

Bel7402-DR cells were seeded in 6-well plates (20,000 cells per well) and then incubated with the NPs(siMALAT1/Pt) at a siRNA dose of 30 nM for 24 h. The cells were then washed with PBS (pH 7.4) solution and further incubated in fresh medium. At predetermined intervals, the cell viability was examined by AlamarBlue assay according to the manufacturer’s protocol. After each measurement, the AlamarBlue agent was removed and the cells were continuously incubated in fresh medium to observe the clone formation.

### Animals

Healthy female BALB/c normal mice and nude mice (4–5 weeks old) were purchased from the Sun Yat-Sen University Experimental Animal Center. All *in vivo* studies were performed in accordance with a protocol approved by the Institutional Animal Care and Use Committee at Sun Yat-Sen University.

### Pharmacokinetics study

Healthy female BALB/c mice were randomly divided into two groups (n = 3) and given an intravenous injection of either (i) naked Cy5-siMALAT1, or (ii) NPs(Cy5-siMALAT1/Pt) at a siRNA dose of 1 nmol per mouse. At predetermined time intervals, orbital vein blood (20 µL) was withdrawn and the fluorescence intensity of Cy5-siMALAT1 in the blood was determined by a microplate reader.

### Xenograft tumor model

Bel7402-DR xenograft tumor model was constructed by subcutaneous injection with 200 μL of Bel7402-DR cell suspension (a mixture of DMEM medium and Matrigel in 1:1 volume ratio) with a density 1 × 10^7^ cells/mL into the back region of healthy female nude mice. When the tumor volume reached 70–100 mm^3^, the mice were used for the following *in vivo* experiments.

### Biodistribution

Bel7402-DR xenograft tumor-bearing mice were randomly divided into two groups (n = 3) and given an intravenous injection of either (i) naked Cy5-siMALAT1, or (ii) NPs(Cy5-siMALAT1/Pt) at a siRNA dose of 1 nmol per mouse and/or 5 mg/kg cisplatin. Twenty-4 hours after the injection, the mice were imaged using a Perkin-Elmer Imaging System. Organs and tumors were then harvested and imaged. To quantify the accumulation of Cy5-siMALAT1, the fluorescence intensity of each tissue was quantified by ImageJ.

### *In Vivo* LncMALAT1 silencing

Bel7402-DR xenograft tumor-bearing mice were randomly divided into four groups (n = 3) and given an intravenous injection of either (i) naked siMALAT1, (ii) NPs(siMALAT1), (iii) NPs(siCTL/Pt) or (iv) NPs(siMALAT1/Pt) at a siRNA dose of 1 nmol per mouse and/or 5 mg/kg cisplatin. After three daily injections, the mice were sacrificed at 24 h post the final injection and the tumor tissues were then collected for *in situ* hybridization (ISH) and qRT-PCR analysis of lncMALAT1 expression.

### Inhibition of xenograft tumor growth

Bel7402-DR xenograft tumor-bearing mice were randomly divided into five groups (n = 5) and intravenously injected with either (i) PBS, (ii) free cisplatin, (iii) NPs(siMALAT1), (iv) NPs(siCTL/Pt), or (v) NPs(siMALAT1/Pt) once every 2 days at a siRNA dose of 1 nmol per mouse and/or 5 mg/kg cisplatin. All the mice were administrated by three consecutive injections and the tumor growth was monitored every 2 days by measuring perpendicular diameters using a caliper and tumor volume was calculated as follows:

$$V=W^{2}\times L/2$$

where W and L are the shortest and longest diameters, respectively.

### Statistical analysis

The *in vitro* data were presented as mean ± S.D. of three independent experiments. Statistical significance was determined by a two-tailed Student’s t*-*test assuming equal variance. A *p*-value <0.05 is considered statistically significant.

## Results and Discussion

### Preparation and characterizations of Co-Delivery NPs

**FIGURE 2:**
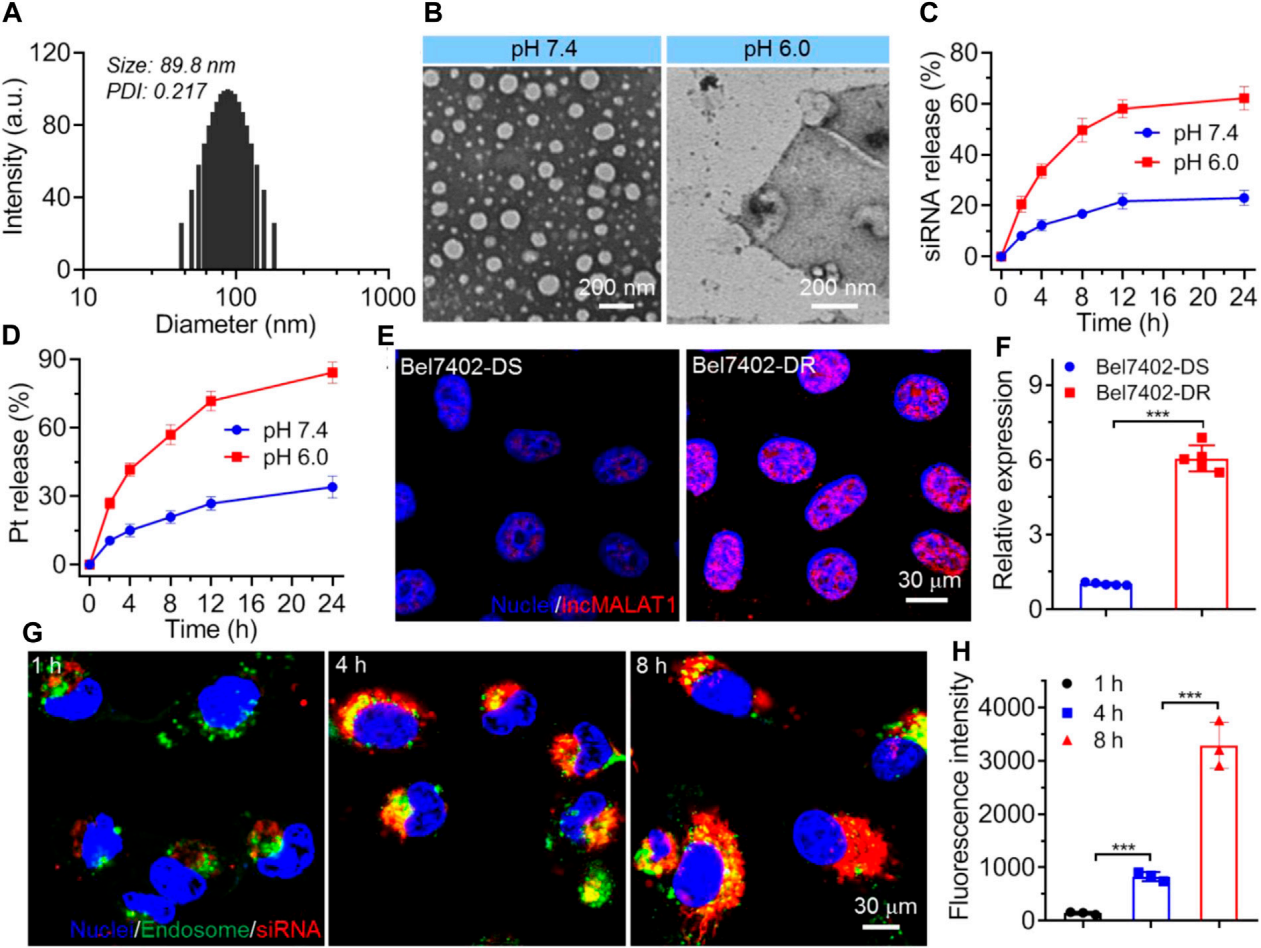
**(A)** Size distribution of NPs(siMALAT1/Pt) incubated in aqueous solution at pH 7.4. **(B)** Morphology of NPs(siMALAT1/Pt) incubated in aqueous solution at pH 7.4 or 6.0. **(C, D)** Cumulative release of Cy5-siMALAT1 **(C)** and cisplatin prodrug **(D)** from the NPs(Cy5-siMALAT1/Pt) incubated in aqueous solution at pH 7.4 or 6.0. **(E, F)** LncMALAT1 expression in Bel7402-DS and Bel7402-DR cells determined by FISH **(E)** and qRT-PCR **(F)**, respectively. **(G)** CLSM images of Bel7402-DR cells incubated with the NPs(Cy5-siMALAT1/Pt) at a siRNA dose of 30 nM and cisplatin dose of 36 mg/L for different times. **(H)** Fluorescence intensity of Cy5-siMALAT1 in the nuclei of Bel7402-DR cells incubated with the NPs(Cy5-siMALAT1/Pt) at a siRNA dose of 30 nM and cisplatin dose of 36 mg/L for different times. ***p < 0.001.

The co-delivery NPs were prepared using the modified nanoprecipitation method (Valencia et al., 2010; Li et al., 2020). In this method, the endosomal pH-responsive copolymer Meo-PEG-*b*-PDPA (Supplementary Figure S1-S3), cisplatin prodrug (Supplementary Figure S4), and the amphiphilic peptide NTPA (C_17_H_35_CONH-PKKKRKVRRRR-OH, Figure 1) were dissolved in DMF and then mixed with the aqueous solution of siMALAT1, which could then spontaneously self-assemble into core-shell NPs (*i.e.*, NPs(siMALAT1/Pt)) with cisplatin prodrug and the complexes of NTPA/siMALAT1 encapsulated into their hydrophobic PDPA cores. As shown in Figure 2A, the NPs(siMALAT1/Pt) are well-dispersed in aqueous solution with an average hydrodiameter of ∼90 nm and a polydispersity (PDI) of 0.217. Further morphological observations show that these co-delivery NPs have a uniform spherical morphology (Figure 1B). At physiological pH (*i.e.*, pH 7.4), these co-delivery NPs show good stability (Supplementary Figure S5). In contrast, because the hydrophobic PDPA segment of copolymer Meo-PEG-*b*-PDPA could be protonated at a pH below its p*K*_*a*_ (∼6.32, Supplementary Figure S6) (Zhou et al., 2011; Xu et al., 2016; Xu et al., 2017), the nanostructure of NPs(siMALAT1/Pt) could be destroyed at an endosomal pH (*e.g.*, pH 6.0) and only a tiny amount of small size NPs that possibly correspond to the NTPA/siMALAT1 complexes could be observed in the aqueous solution at pH 6.0 (Figure 2B). This endosomal pH-responsive characteristic is further proved by the release kinetics of loaded siMALAT1 and cisplatin prodrug. As displayed in Figures 2C, D, with the nanostructure disassociation at an endosomal pH, the NPs(siMALAT1/Pt) could rapidly release their loaded siMALAT1 (Figure 2C) and cisplatin prodrug (Figure 2D) at pH 6.0.

We next evaluated whether the NPs(siMALAT1/Pt) could employ their endosomal pH-responsive characteristic to enhance the endosomal escape and nucleus entry of loaded siMALAT1. To this end, cisplatin-resistant Bel7402 cells (*i.e.*, Bel7402-DR) and their parental cell line (*i.e.*, Bel7402-DS) were chosen (Supplementary Figure S7), as lncMALAT1 expression is significantly upregulated in Bel7402-DR cells (Figures 2E, F) and accumulating evidences have demonstrated the important contribution of high lncMALAT1 expression to the resistance to various chemotherapeutic drugs including cisplatin (Schmitt and Chang, 2016; Hou et al., 2023). As shown in Figure 2G, the NPs(siMALAT1/Pt) could be internalized by Bel7402-DR cells and a number of loaded siRNA molecules could escape from the endosomes within 4 h incubation. When prolonging the incubation time to 8 h, these escaped siRNA molecules could enter the nuclei, as revealed by the co-localization between red and blue fluorescence. This result is further proven by the detection of siMALAT1 fluorescence in the isolated nuclei (Figure 2H), in which the fluorescence intensity of siMALAT1 in the nuclei of Bel7402-DR cells increases by more than 4-fold when prolonging the incubation time from 4 to 8 h. If replacing the nucleus-targeting peptide NTPA with the cationic lipid-like compound G0-C14 (Supplementary Figure S8) we previously developed (Li et al., 2020; Yang et al., 2023), the corresponding NPs (*i.e.*, Control NPs) could be also internalized by Bel7402-DR cells and the loaded siMALAT1 molecules could efficiently escape from the endosomes, which however are predominately localized in the cytoplasm (Supplementary Figure S9). These findings strongly demonstrate the importance of NTPA to the NPs(siMALAT1/Pt)-mediated nucleus-specific delivery of siRNA.

### *In Vitro* LncMALAT1 silencing and reversal of cisplatin resistance

**FIGURE 3:**
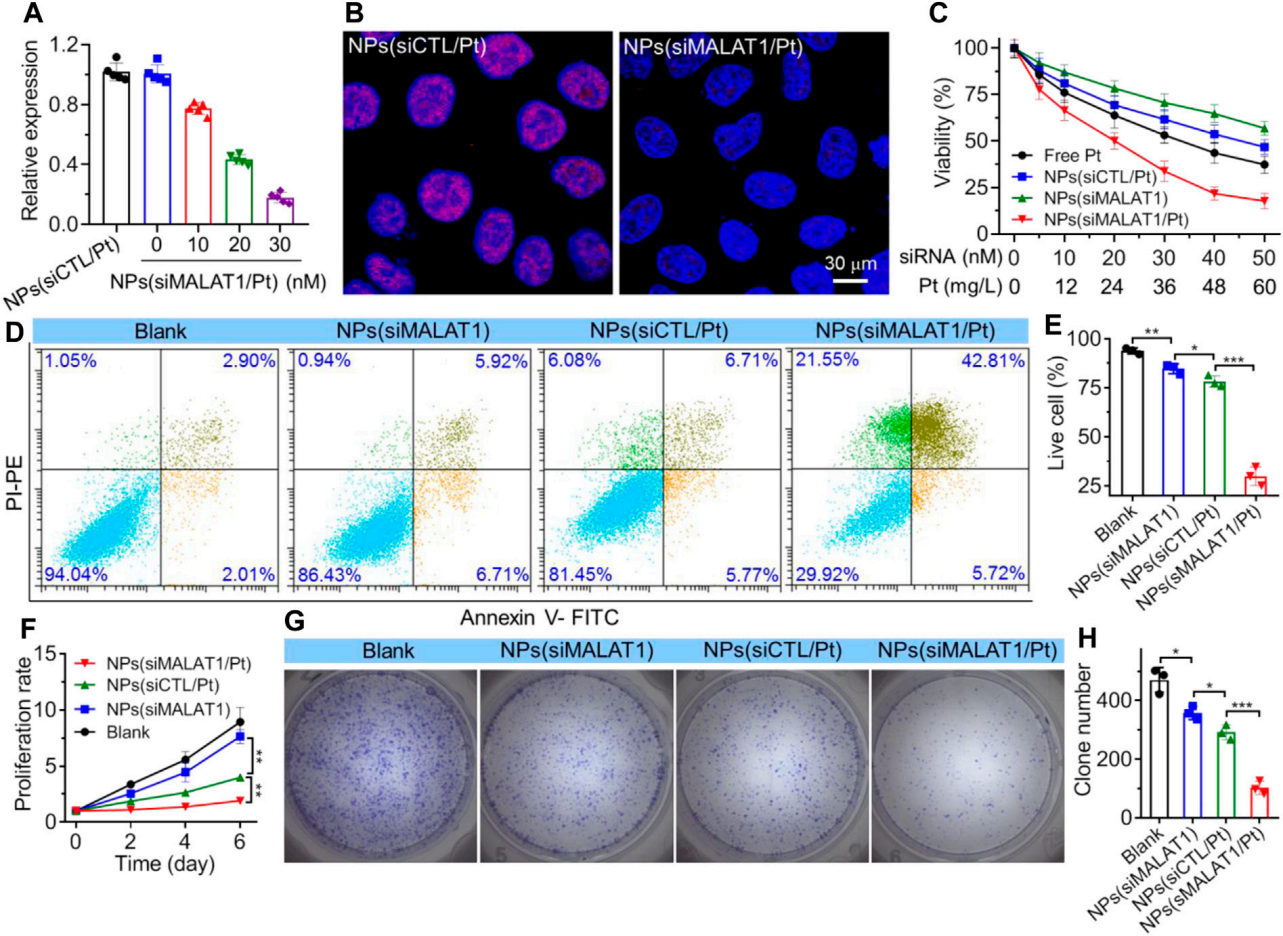
**(A)** qRT-PCR analysis of lncMALAT1 expression in Bel7402-DR cells treated with the NPs(siMALAT1/Pt) at different doses of siRNA and cisplatin. **(B)** FISH analysis of lncMALAT1 expression in Bel7402-DR cells treated with the NPs(siMALAT1/Pt) at a siRNA dose of 30 nM and cisplatin dose of 36 mg/L. **(C)** Viability of Bel7402-DR cells treated with the NPs(siMALAT1/Pt) at different doses of siRNA and cisplatin. **(D, E)** Flow cytometry analysis **(D)** and the corresponding statistical results **(E)** of apoptosis of Bel7402-DR cells respectively treated with the NPs(siMALAT1), NPs(siCTL/Pt), and NPs(siMALAT1/Pt) at a siRNA dose of 30 nM and/or cisplatin dose of 36 mg/L. Blank represents the cells incubated in DMEM medium containing 10% FBS. **(F–H)** Proliferation profile and clone formation **(G)** as well as the corresponding statistical results **(H)** of Bel7402-DR cells treated with the formulas shown in **(D)**. *p < 0.05; **p < 0.01; ***p < 0.001.

After validating the nucleus-specific delivery of siRNA by NPs(siMALAT1/Pt), we next investigated whether these NPs could silence lncMALAT1 expression and reverse the cisplatin resistance. As shown in Figure 3A, compared to the NPs loading scramble siRNA and cisplatin prodrug (*i.e.*, NPs(siCTL/Pt)), the NPs(siMALAT1/Pt) could efficiently down-regulate lncMALAT1 expression in Bel7402-DR cells and more than 80% of lncMALAT1 could be downregulated at a siMALAT1 concentration of 30 nM. The similar result could be also found in the FISH analysis of Bel7402-DR cells (Figure 3B), in which the expression of lncMALAT1 stained with red fluorescence is significantly down-regulated. With this efficient lncMALAT1 silencing, the sensitivity of Bel7402-DR cells to cisplatin is apparently improved. As displayed in Figure 3C, the viability of Bel7402-DR cells is higher than 60% after 24 h treatment with the NPs(siMALAT1) or NPs(siCTL/Pt) at a siRNA dose of 30 nM or cisplatin dose of 36 mg/L. However, the cell viability is ∼30% after 24 h treatment with the NPs(siMALAT1/Pt) at a siRNA dose of 30 nM and cisplatin dose of 36 mg/L, and the corresponding half-maximal inhibitory concentration (IC_50_) value is ∼23.8 mg/L, which is much lower than that of free cisplatin (∼43.6 mg/L). This enhanced cisplatin sensitivity is further demonstrated by the flow cytometry analysis of apoptosis of Bel7402-DR cells. As presented in Figures 3D, E, the apoptosis rate of Bel7402-DR cells reaches ∼48% and less than 30% of cells are alive after 24 h treatment with the NPs(siMALAT1/Pt) at a siRNA dose of 30 nM and cisplatin dose of 36 mg/L. In contrast, more than 80% of Bel7402-DR cells are alive after 24 h treatment with the NPs(siMALAT1), NPs(siCTL/Pt) or free cisplatin at the same conditions. Due to this reversal of cisplatin resistance and enhanced cisplatin sensitivity, the proliferation of Bel7402-DR cells is significantly suppressed (Figures 3F–H). Compared to the cells treated with other formulas, Bel7402-DR cells treated with the NPs(siMALAT1/Pt) show much weaker ability to proliferate (Figure 3F) and form clones (Figures 3G, H). All these results clearly indicate that the NPs(siMALAT1/Pt) could reverse the cisplatin resistance of Bel7402-DR cells and thus enhance their sensitivity to cisplatin via efficient silencing of lncMALAT1 expression.

### *In Vivo* LncMALAT1 silencing and anticancer effect

**FIGURE 4:**
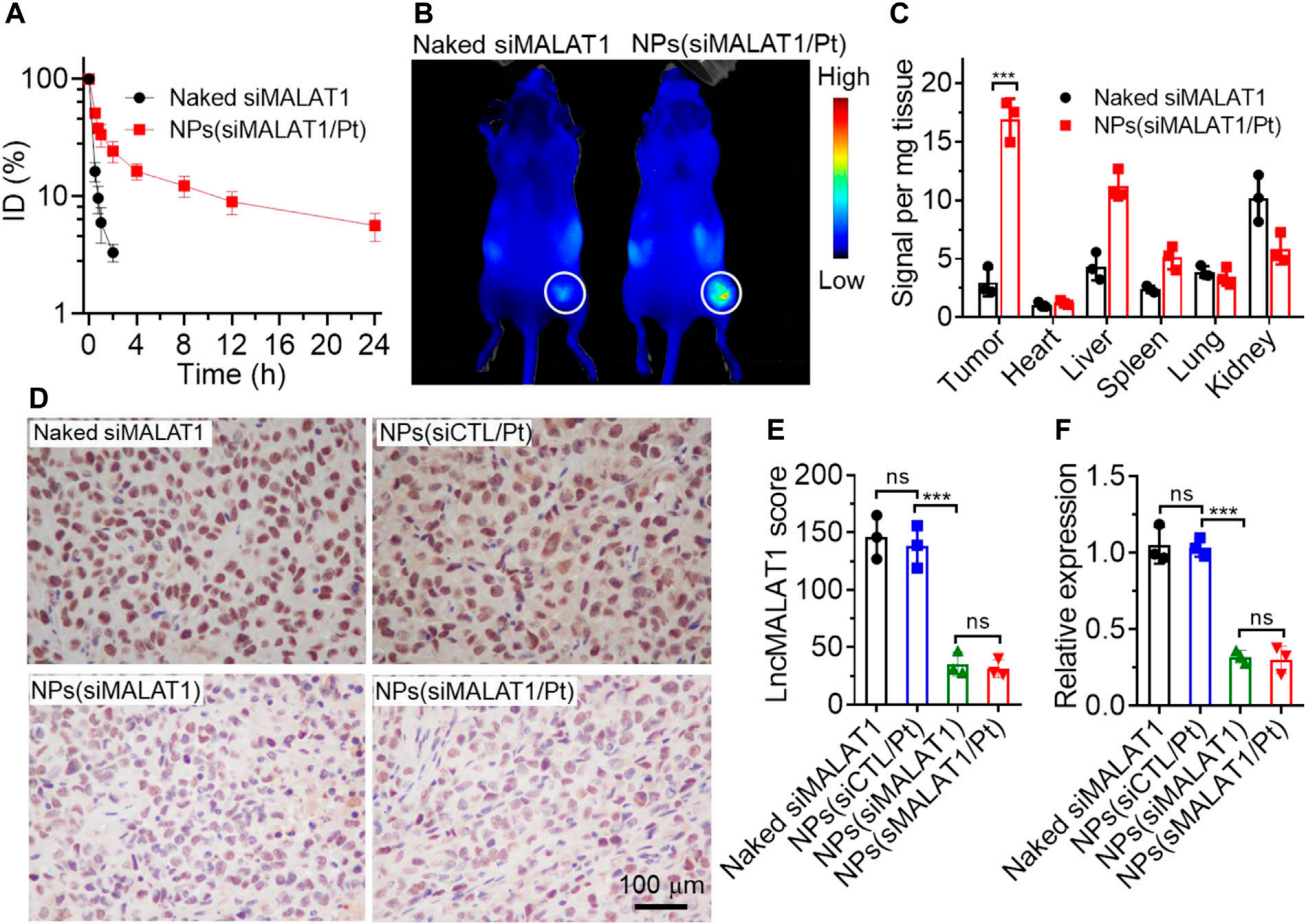
**(A)** Blood circualtion profile of naked Cy5-siMALAT1 and NPs(Cy5-siMALAT1/Pt). **(B)** Overlaid fluorescent image of Bel7402-DR xenograft tumor-bearing mice at 24 h post injection of naked Cy5-siMALAT1 and NPs(Cy5-siMALAT1/Pt). **(C)** Biodistribution of naked Cy5-siMALAT1 and NPs(Cy5-siMALAT1/Pt) in the tumors and major organs of Bel7402-DR xenograft tumor-bearing mice sacrificed at 24 h post injection. **(D, E)** ISH analysis **(D)** and the corresponding statistical results **(E)** of lncMALAT1 expression in the tumor tissues of Bel7402-DR xenograft tumor-bearing mice treated with naked siMALAT1, NPs(siMALAT1), NPs(siCTL/Pt), and NPs(siMALAT1/Pt), respectively. **(F)** qRT-PCR analysis of lncMALAT1 expression in the tumor tissues of Bel7402-DR xenograft tumor-bearing mice treated with the formulas shown in **(D)**. *ns*, no significance; ***p < 0.001.

Having validated the ability of NPs(siMALAT1/Pt) to efficiently reverse cisplatin resistance via silencing lncMALAT1 expression *in vitro*, we next evaluated their ability to silence lncMALAT1 expression *in vivo* and inhibit the cisplatin-resistant tumor growth. To do this, the pharmacokinetics of NPs(siMALAT1/Pt) was first examined by intravenous injection of these NPs into healthy mice. As shown in Figure 4A, due to the protection of PEG outer shell (Knop et al., 2010), the NPs(siMALAT1/Pt) could significantly prolong the blood circulation capability of siMALAT1 and around 10% of loaded siMALAT1 could be still detected in the blood at 12 h post injection. In contrast, almost all the naked siMALAT1 molecules have been cleared from the blood at 2 h post injection. Because of this prolonged blood circulation, the siMALAT1 molecules encapsulated in the NPs(siMALAT1/Pt) show more than 7-fold higher accumulation in Bel7402-DR xenograft tumor tissues compared to naked siMALAT1 (Figures 4B, C). With these encouraging results, we subsequently intravenously injected the NPs(siMALAT1/Pt) into Bel7402-DR xenograft tumor-bearing mice and assessed their ability to silence lncMALAT1 expression *in vivo*. As displayed in Figures 4D, E, the NPs(siMALAT1/Pt) could significantly downregulate lncMALAT1 expression, as proven by the much lower lncMALAT1 expression score compared to naked siMALAT1 or NPs(siCTL/Pt). This efficient lncMALAT1 silencing is further proven by the qRT-PCR analysis of lncMALAT1 expression in the tumor tissues (Figure 4F), in which the NPs(siMALAT1/Pt) could down-regulate lncMALAT1 expression by around 70%.

**FIGURE 5:**
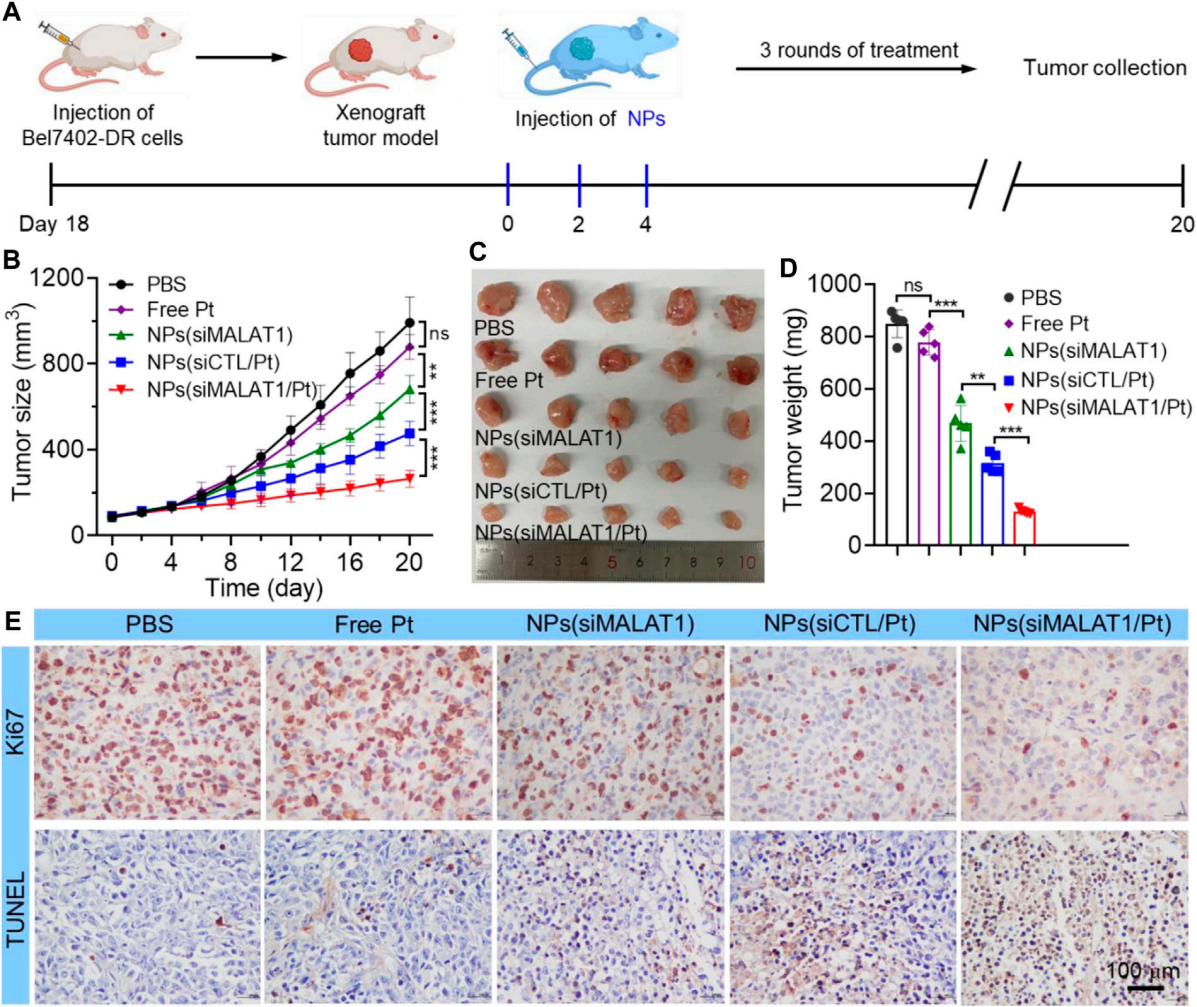
**(A)** Schematic illustartion of tumor inoculation and treatment of Bel7402-DR xenograft tumor-bearing mice with PBS, free cisplatin, NPs(siMALAT1), NPs(siCTL/Pt), and NPs(siMALAT1/Pt), respectively. **(B–D)** Tumor growth **(B)**, image of collected tumors **(C)**, and tumor weight **(D)** of Bel7402-DR tumor-bearing mice after systemic treatment in each group. **(E)** Ki67 and TUNEL staining of tumor tissues of Bel7402-DR tumor-bearing mice after systemic treatment in each group. *ns*, no significance; **p < 0.01; ***p < 0.001.

We finally investigated the anticancer effect of NPs(siMALAT1/Pt) via intravenous injection of these NPs into Bel7402-DR xenograft tumor-bearing mice (Figure 5A). As expected, the administration of NPs(siMALAT1/Pt) could efficiently inhibit the tumor growth and there is less than 3-fold increase in the tumor size within an evaluation period of 20 days (Figures 5B–D). The NPs(siMALAT1) or NPs(siCTL/Pt) could also inhibit the tumor growth since they have the ability to silence lncMALAT1 expression or transport cisplatin prodrug in tumor cells. However, their tumor inhibition efficacy is much weaker than that of NPs(siMALAT1/Pt) showing the characteristic of reversing cisplatin resistance via silencing of lncMALAT1 expression. The much stronger anticancer effect of NPs(siMALAT1/Pt) than other therapeutic formulas could be also found in the results of histological analysis (Figure 5E), in which less proliferation represented by Ki67 staining and more apoptosis labeled by TUNEL staining could be observed in the tumor tissues of mice treated with the NPs(siMALAT1/Pt). Notably, the administration of NPs(siMALAT1/Pt) does not induce the decrease in the mouse body weight (Supplementary Figure S10) and apparent histological change in the main organs (Supplementary Figure S11). All these results imply the low *in vivo* toxicity of NPs(siMALAT1/Pt) developed in this work.

## Conclusion

In summary, we have developed an endosomal pH-responsive nanoplatform to co-deliver siRNA and cisplatin prodrug for effective cisplatin-resistant HCC therapy. This co-delivery nanoplatform shows a long blood circulation and high accumulation in the tumor tissues. After internalization by tumor cells, this nanoplatform could employ its endosomal pH-responsive characteristic to enhance the endosomal escape of loaded cisplatin prodrug and NTPA/siMALAT1 complexes, which could specifically transport siMALAT1 into the nuclei and significantly silence lncMALAT1 expression, leading to the reversal of cisplatin resistance and efficient inhibition of cisplatin-resistant HCC tumor growth. The co-delivery nanoplatform developed herein shows the potential as an effective tool for cancer treatment.

## Data Availability

The original contributions presented in the study are included in the article/Supplementary Material, further inquiries can be directed to the corresponding authors.
