# Supplementary material for: Nanoparticles (NPs)-mediated lncMALAT1 silencing to reverse cisplatin resistance for effective hepatocellular carcinoma therapy
Source: Front Pharmacol. 2024 Jul 30;15:1437071. doi: 10.3389/fphar.2024.1437071 (PMC11319142; doi:10.3389/fphar.2024.1437071)
Supplement: Supplementary file 1 [file DataSheet1.docx]

Supporting Information

*of*

**Nanoparticles (NPs)-mediated lncMALAT1 silencing to reverse cisplatin resistance for effective hepatocellular carcinoma therapy**

*Ting Wang,^1,2^Qianyao Li,^1,2,3^ Rui Xu,^2,3^ Zixuan Zhao,^1,2^ Qi Sun,^4*^ Xiaoding Xu,^2,3^* Rong Li^1^**

*^1^ The Second Affiliated Hospital, Department of Pharmacy, Hengyang Medical School, University of South China, Hengyang 421001, P. R. China*

*^2^ Guangzhou Key Laboratory of Medical Nanomaterials, Sun Yat-Sen Memorial Hospital, Sun Yat-Sen University, Guangzhou 510120, P. R. China*

*^3^ Nanhai Translational Innovation Center of Precision Immunology, Sun Yat-Sen Memorial Hospital, Foshan 528200, P. R. China*

*^4^ Department of Neurosurgery, Yiyang Central Hospital, Yiyang 413000, P. R. China*

*Correspondence: l7979r@163.com; xuxiaod5@mail.sysu.edu.cn; sunqiqw@163.com

**1. Materials**

Methoxyl-polyethylene glycol (Meo-PEG_113_-OH) was purchased from JenKem Technology. 2-(Diisopropyl amino) ethyl methacrylate (DPA-MA), α-bromoisobutyryl bromide, tetrahydrofuran (THF), triethylamine (TEA), *N,N,N',N',N'*-pentamethyldiethylenetriamine (PMDETA), isopropyl alcohol, copper (I) bromide (CuBr), *N,N'*-dimethylformamide (DMF), dimethyl sulfoxide (DMSO), *cis,trans,cis*-[PtCl_2_(OH)_2_(NH_3_)_2_] (DDP), sebacic anhydride, and dichloromethane (DCM) were acquired from Sigma-Aldrich and used directly. Cationic lipid-like compound alkyl-modified polyamidoamine (PAMAM) dendrimer (G0-C14) was synthesized through ring opening of 1,2-epoxytetradecane by generation 0 of (PAMAM) dendrimer according to our previous studies [1, 2]. The GSH-responsive cisplatin prodrug was synthesized via the reaction between DDP and sebacic anhydride according to previous report [3].

**2. Synthesis of Meo-PEG-Br**

Meo-PEG_113_-OH (8 g, 1.6 mmol) and TEA (1.3 mL, 9.6 mmol) were dissolved in 250 mL of DCM. In an ice-salt bath, α-bromoisobutyryl bromide (l mL, 8 mmol) dissolved in 10 mL of DCM was added dropwise. After stirring for 24 h, the mixture was washed with 1 M NaOH (3 × 50 mL), 1 M HCl (3 × 50 mL), and deionized water (3 × 50 mL), respectively. After drying over anhydrous MgSO4, the solution was concentrated, and cold ether was added to precipitate the product. After re-precipitation thrice, the product was collected as white powder after drying under vacuum. The synthesis of Meo-PEG-Br is shown in Figure S2. The ^1^HNMR spectrum of Meo-PEG-Br is shown in Figure S2.

**3. Synthesis of methoxyl-polyethylene glycol-*b*-poly (2-(diisopropylamino) ethylmethacrylate) (Meo-PEG-*b*-PDPA)**

The polymer Meo-PEG-*b*-PDPA was synthesized by atom transfer radical polymerization (ATRP) according to our previous studies [1, 2]. DPA-MA (2.6 g, 12 mmol), Meo-PEG-Br (0.75 g, 0.15 mmol), and PMDETA (31.5 μL, 0.15 mmol) were added to a polymerization tube. DMF (3 mL) and 2-propanol (3 mL) were then added to dissolve the monomer and initiator. After three cycles of freeze-pump-thaw to remove oxygen, CuBr (21.6 mg, 0.15 mmol) was added under nitrogen atmosphere and the polymerization tube was sealed under vacuum. After polymerization at 40 ^o^C for 24 h, tetrahydrofuran (THF) was added to dilute the product, which was then passed through a neutral Al_2_O_3_ column. The resulting THF suspension was concentrated and the residue was dialyzed against THF, followed by deionized water. The polymer was collected as a white powder after freeze-drying under vacuum. The synthesis of Meo-PEG-b-PDPA is shown in Figure S3. The ^1^HNMR spectrum is shown in Figure S4. The molecular weight was determined by gel permeation chromatography (GPC) using THF as eluent. *M_n, GPC_* = 2.37 × 10^4^ (PDI = 1.22); *M_n,NMR_* = 2.21 × 10^4^.

**4. ^1^H Nuclear magnetic resonance (^1^HNMR)**

The ^1^HNMR spectra of the polymers were recorded on a Mercury VX-300 spectrometer at 400 MHz (Varian, USA), using CDCl_3_ as a solvent and TMS as an internal standard.

**5. Gel permeation chromatography (GPC)**

Number- and weight-average molecular weights (*M_n_* and *M_w_*, respectively) of Meo-PEG-*b*-PDPA polymer were determined by gel permeation chromatographic system equipped with a Waters 2690D separations module and a Waters 2410 refractive index detector. THF was used as the eluent at a flow rate of 0.3 mL/min. Waters millennium module software was used to calculate molecular weight based on a universal calibration curve generated by polystyrene standard of narrow molecular weight distribution.

**6. Acid-base titration**

The polymer Meo-PEG-*b*-PDPA was dispersed in deionized water, and a concentrated HCl aqueous solution was added until the copolymer was completely dissolved (1 mg/mL). Subsequently, 1 M NaOH aqueous solution was added in 1-5 μL increments. After each addition, the solution was constantly stirred for 3 min, and the solution pH was measured using a pH meter. The *pK_a_* of Meo-PEG-*b*-PDPA polymer was determined as the pH at which 50% of polymer turns ionized.

**7. *In vitro* drug release**

The NPs(Cy5-siMALAT1/Pt) were dispersed in 1 mL of PBS (pH 7.4) and then transferred to a Float-a-lyzer G2 dialysis device (MWCO 100 kDa, Spectrum) that was immersed in PBS (pH 7.4 or 6.0) at 37 ^o^C. At a predetermined interval, 5 μL of the NP solution was withdrawn and mixed with 20-fold DMSO. The fluorescence intensity of Cy5-siMALAT1 was determined by Synergy HT multi-mode microplate reader. At the same time, the mixture was subjected for inductively coupled plasma mass spectrometry (ICP-MS) analysis of platinum content

**8. qRT-PCR**

Total RNA was extracted from the cultured cells using Trizol and 1 μg of RNA was then reverse transcribed into cDNAs using a Superscript First-Strand cDNA Synthesis Kit (18080-051, Invitrogen, USA). qRT-PCR analysis was performed using SYBR Premix Ex Taq II kit (DRR081A, TAKARA, Japan) on a LightCycler 480 System (Roche, Switzerland).

**9.** **Histology**

Healthy female BALB/c mice were randomly divided into five groups (n = 3) and administered daily intravenous injections of either (i) PBS, (ii) free cisplatin, (iii) NPs(siMALAT1), (iv) NPs(siCTL/Pt), or (v) NPs(siMALAT1/Pt) at a siRNA dose of 1 nmol per mouse and/or 5 mg/kg cisplatin. After three consecutive injections, the main organs were collected 24 h post the final injection, fixed with 4% paraformaldehyde, and embedded in paraffin. Tissue sections were stained with hematoxylin-eosin (H&E) and then viewed under an optical microscope.

**10. Immunohistochemistry (IHC) staining**

IHC staining was performed on formalin-fixed paraffin-embedded tumor sections. In brief, tumor slides were first heated to 60 °C for 1 h, desparaffinized with xylene, and washed with different concentrations of alcohol. After retrieval of antigen using DAKO target retrieval solution at 95-99 °C for 40 min, followed by washing, the slides were blocked with peroxidase blocking buffer (DAKO Company) for 5 min. After washing buffer (DAKO Company), the slides were incubated with the primary antibody diluted in DAKO antibody solution for 1 h. The slides were then washed and incubated with peroxidase-labeled polymer for 30 min. After washing and staining with DAB+ substrate-chromogen solution and hematoxylin, the slides that remounted and viewed under an Olympus microscope.

**References**

[1] Yang, K., Xu, L., Xu, Y., Shen, Q., Qin, T., Yu, Y., Nie, Y., Yao, H., Xu, X. *Acta Pharm. Sin. B* **2023**, 13, 3489-3502.

[2] Li, Q., Qin, T., Bi, Z., Hong, H., Ding, L., Chen, J., Wu, W., Lin, X., Fu, W., Zheng, F., Yao, Y., Luo, M.-L., Saw, P.E., Wulf, G.M., Xu, X., Song, E., Yao, H., Hu, H. *Nat. Commun.* **2020**, 11, 1456..

[3] Xu, X.; Xie, K.; Zhang, X. Q.; Pridgen, E. M.; Park, G. Y.; Jinjun Shi, J.; Wu, J.; Kantoff, P. W.; Lippard, S. J.; Langer, R.; Walker, G. C.; Farokhzad, O. C. *Proc. Natl. Acad. Sci. USA* **2013**, 110, 18638-18643.





**Figure S1.** Synthesis route of the polymer Meo-PEG-Br and Meo-PEG-*b*-PDPA.





**Figure S2.** ^1^HNMR spectrum of the polymer Meo-PEG-Br in CDCl_3_.





**Figure S3.** ^1^HNMR spectrum of the polymer Meo-PEG-*b*-PDPA in CDCl_3_.

**
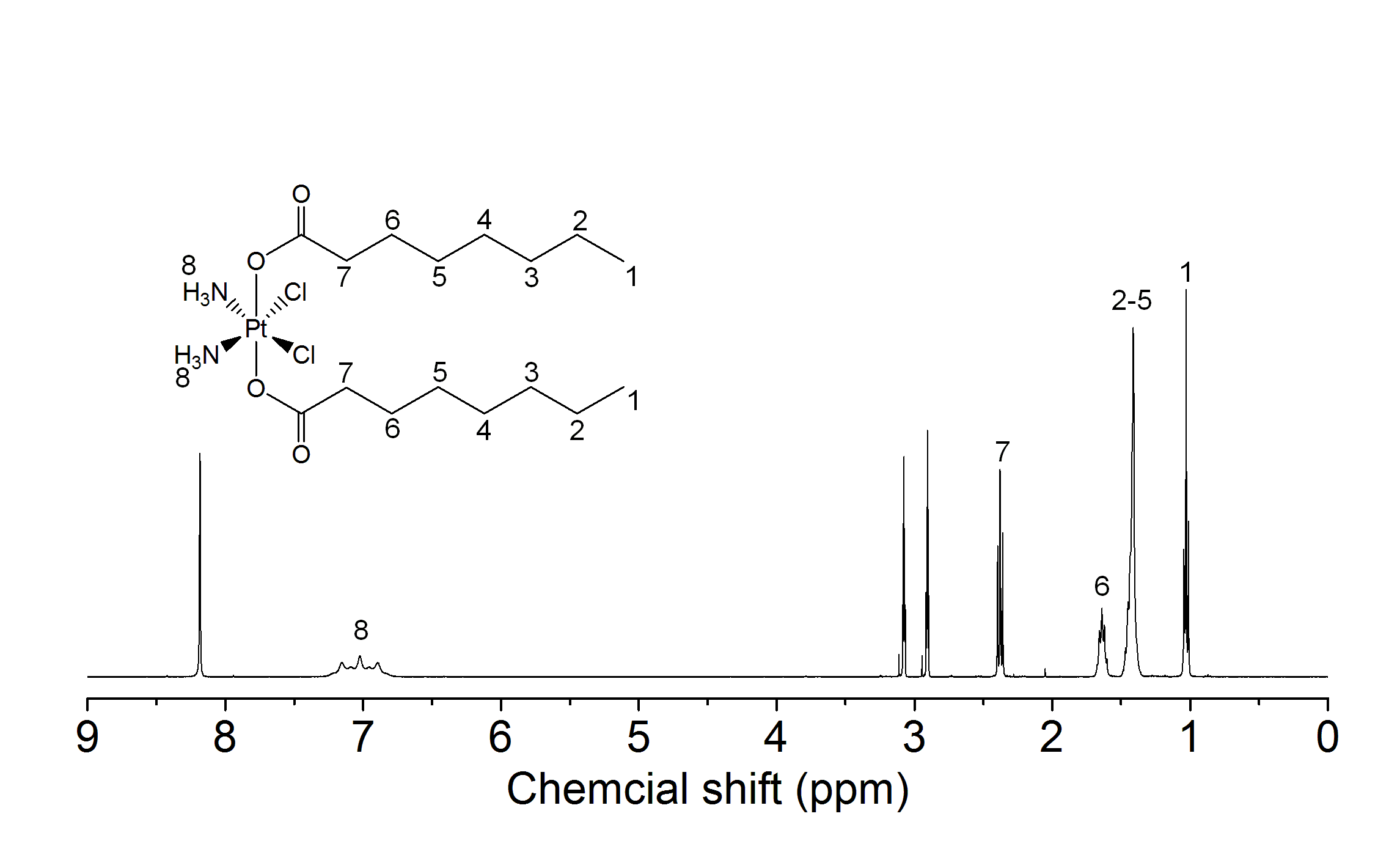
**

**Figure S4.** ^1^HNMR spectrum of the GSH-responsive cisplatin prodrug in CDCl_3_.


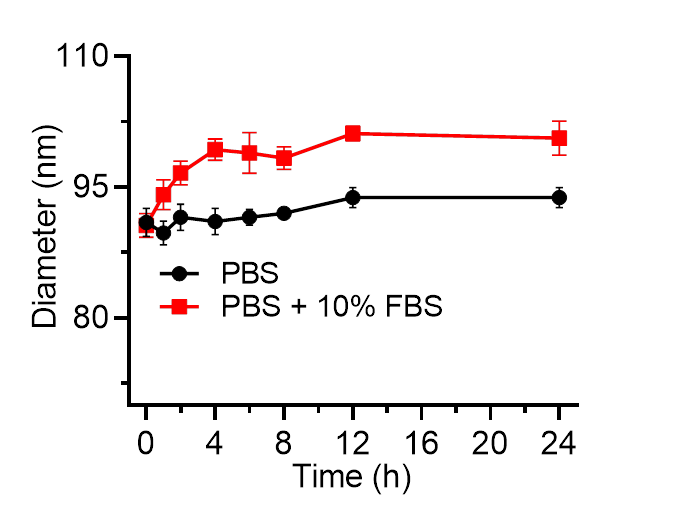


**Figure S5.** Size change of the NPs(siMALAT1/Pt) incubated in PBS and FBS-containing PBS solution for different times.





**Figure S6.** Acid-base titration profile of the polymer Meo-PEG-*b*-PDPA.


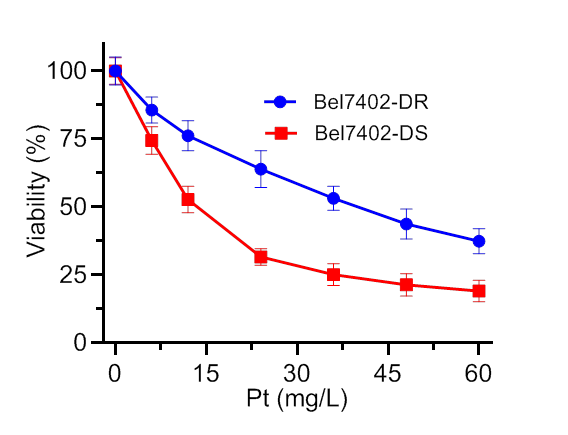


**Figure S7.** Viability of Bel7402-DS and Bel7402-DR cells treated with cisplatin at different concentrations for 24 h.


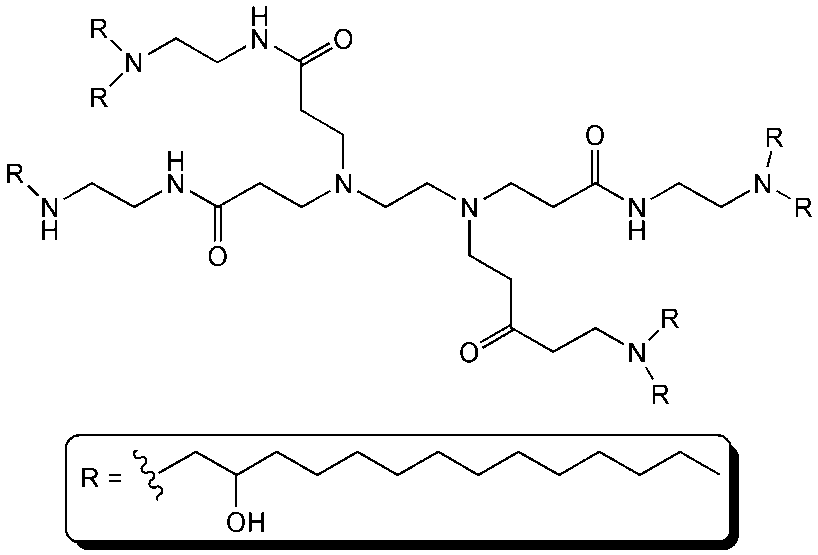


**Figure S8.** Chemical structure of the amphiphilic cationic lipid G0-C14.


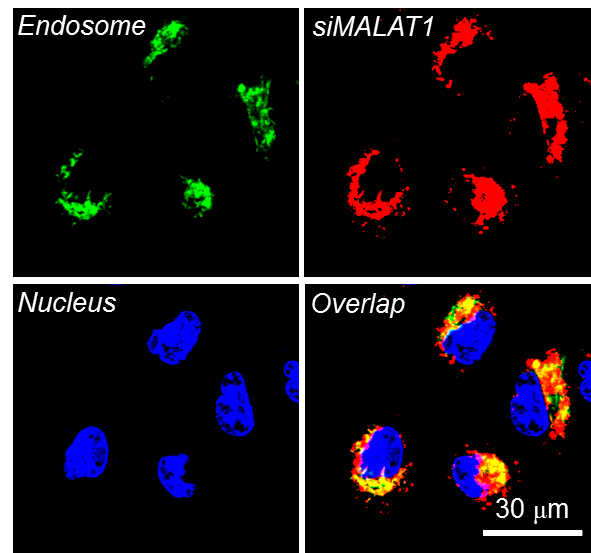


**Figure S9.** Fluorescent images of Bel7402-DR cells incubated with the Control NPs for 8 h.


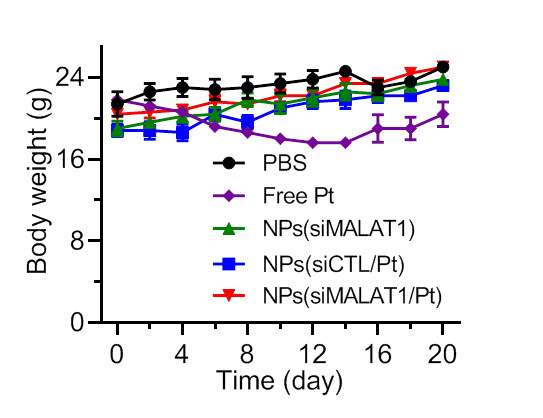


**Figure S10.** Body weight of Bel7402-DR xenograft tumor-bearing mice with PBS, free cisplatin, NPs(siMALAT1), NPs(siCTL/Pt), and NPs(siMALAT1/Pt), respectively.


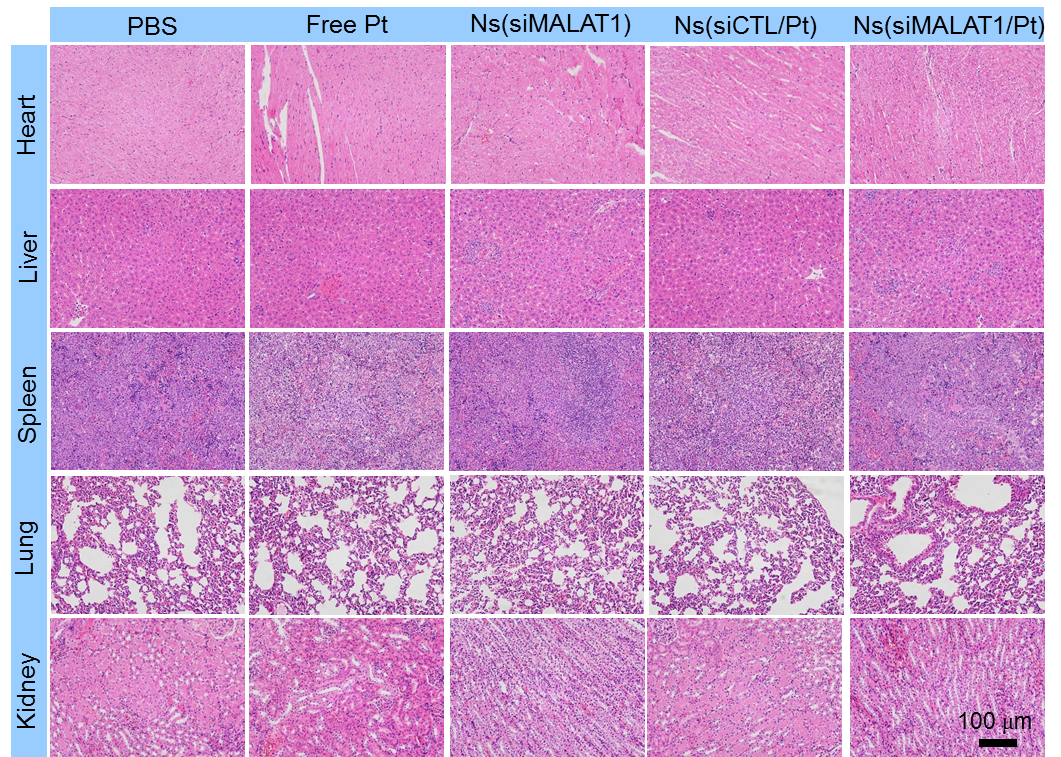


**Figure S11.** Histological analysis of major organs of healthy mice received three consecutive intravenous injections of PBS, free cisplatin, NPs(siMALAT1), NPs(siCTL/Pt), and NPs(siMALAT1/Pt), respectively.
